# Baseline omega-3 nutritional status and supplementation response in pediatric attention-deficit/hyperactivity disorder: a systematic review and biomarker-stratified meta-analysis

**DOI:** 10.3389/fpubh.2026.1844881

**Published:** 2026-06-22

**Authors:** Xiao Fu, Ling Feng, Mingyan Jiang, Jinrong Li

**Affiliations:** 1 Department of Pediatrics, West China Second University Hospital, Sichuan University, Chengdu, China; 2 Key Laboratory of Birth Defects and Related Diseases of Women and Children, Ministry of Education, Sichuan University, Chengdu, China

**Keywords:** attention-deficit/hyperactivity disorder, biomarker, docosahexaenoic acid, eicosapentaenoic acid, meta-analysis, neurodevelopment, omega-3 polyunsaturated fatty acids, precision nutrition

## Abstract

**Introduction:**

High heterogeneity characterizes the efficacy of omega-3 polyunsaturated fatty acid (PUFA) supplementation for attention-deficit/hyperactivity disorder (ADHD). This study aimed to examine whether baseline omega-3 nutritional status may partly explain variability in supplementation response.

**Methods:**

We conducted a PRISMA-compliant systematic review and meta-analysis registered in PROSPERO (CRD420251218861). PubMed, Embase, PsycINFO, and CENTRAL were searched from inception to December 2025. Eligible studies were randomized controlled trials (RCTs) of omega-3 supplementation in children and adolescents with ADHD that reported baseline nutritional biomarkers, such as erythrocyte EPA/DHA, and efficacy outcomes stratified or otherwise separable according to baseline biomarker status. Data were synthesized using random-effects meta-analysis of standardized mean differences (SMDs), with a prespecified categorical subgroup analysis comparing low-baseline versus normal/high or unstratified baseline estimates.

**Results:**

Seven RCTs, contributing 10 analytical estimates, were included. The overall pooled analysis showed a small statistically significant effect of omega-3 supplementation (SMD = 0.21, 95% CI: 0.04–0.38; *p*= 0.017), with moderate heterogeneity (*I*^2^ = 38.7%). Stratification by baseline biomarkers was associated with lower within-subgroup heterogeneity and suggested differential treatment effects by baseline status (χ^2^ = 10.44, *df* = 1, *p* = 0.0012). The low-baseline subgroup (k = 4) showed a larger estimated effect (SMD = 0.52, 95% CI: 0.28–0.76; *p* < 0.001; *I*^2^ = 0%), whereas the normal/high or unstratified baseline subgroup (*k* = 6) did not show a statistically significant effect (SMD = 0.03, 95% CI: −0.15–0.21; *p* = 0.77; *I*^2^ =).

**Discussion:**

Omega-3 supplementation response in pediatric ADHD may vary according to baseline nutritional status. However, the evidence base remains limited to seven RCTs, and definitions of low baseline status differed across trials according to biomarker matrix and study-specific thresholds. These findings should therefore be interpreted as preliminary and hypothesis-generating. Larger, prospectively stratified RCTs using harmonized biomarker assessments and standardized baseline cut-offs are needed before biomarker-guided omega-3 supplementation can be recommended for clinical nutritional guidance.

**Systematic review registration:**

PROSPERO https://www.crd.york.ac.uk/PROSPERO/view/CRD420251218861, identifier CRD420251218861.

## 1 Introduction

Attention-deficit/hyperactivity disorder (ADHD) is recognized as one of the most prevalent and heterogeneous neurodevelopmental disorders, characterized by a persistent and impairing pattern of inattention, hyperactivity, and impulsivity. Epidemiological data indicate that ADHD affects approximately 5.3%−8.0% of children and adolescents globally, frequently persisting into adulthood and causing functional impairments across academic, social, and familial domains (1–3). While central nervous system stimulants (such as methylphenidate and amphetamine derivatives) and non-stimulant medications (such as atomoxetine and guanfacine) remain the established first-line pharmacotherapies, long-term pharmacological management is frequently limited by parental hesitancy regarding long-term psychotropic medication use in developing brains, a notable incidence of adverse effects (e.g., appetite suppression, sleep disturbances, cardiovascular concerns), and the reality that up to 30% of patients exhibit partial or non-response to conventional treatments (4, 5). Furthermore, while standard pharmacotherapies effectively address the core behavioral and attentional symptoms of ADHD, their impact on associated emotional dysregulation, systemic inflammation, and broader physiological health is often variable or negligible, underscoring a critical need for safe, multimodal, and adjunctive therapeutic strategies (6, 7).

In this context, dietary and nutritional interventions are increasingly investigated, with omega-3 polyunsaturated fatty acids (PUFAs) emerging as the most extensively researched and biologically plausible candidates. While increasing dietary intake of marine fish is the primary natural source of these fatty acids, standardized PUFA supplementation is generally preferred in pediatric clinical trials. Supplements circumvent the poor dietary adherence frequently observed in children with ADHD, avoid the risk of heavy metal accumulation associated with high fish consumption, and ensure the delivery of precise, therapeutic dosages necessary for double-blinded evaluations. The rationale for utilizing long-chain omega-3 PUFAs—specifically eicosapentaenoic acid (EPA) and docosahexaenoic acid (DHA)—is based on their roles in neurodevelopment and brain architecture (8, 9). DHA is selectively concentrated in neuronal membranes, where it governs membrane fluidity, receptor function, and optimal synaptic transmission. Conversely, EPA, though present in lower concentrations in the brain parenchyma, plays a critical regulatory role in neuroinflammation and cerebral blood flow. Together, they modulate the monoaminergic neurotransmitter systems, particularly dopamine and serotonin, which are central to the pathophysiological models of ADHD (8, 9).

Despite this robust mechanistic foundation, the translation from nutritional neuroscience to clinical efficacy remains inconsistent. The existing literature comprises dozens of randomized controlled trials (RCTs) assessing omega-3 supplementation in pediatric ADHD, yet the clinical outcomes are mixed. Previous comprehensive meta-analyses have repeatedly documented only modest overall effect sizes (often ranging from SMD = 0.15 to 0.30) (10–14). These pooled analyses have reported between-study heterogeneity and variability in treatment response across trials (10, 12–15). This discrepancy suggests the presence of unrecognized or inadequately controlled sources of clinical and methodological heterogeneity, including baseline fatty-acid status (15, 16).

We hypothesize that baseline nutritional status, objectively measured via EPA and DHA biomarkers (such as erythrocyte membranes or plasma fractions), may be an important determinant of treatment response (15, 16). Unlike many pharmacological interventions, nutrient supplementation may operate partly through repletion of an insufficient baseline state. Consequently, it is biologically plausible that omega-3 supplementation may yield larger effects in individuals with pre-existing low omega-3 biomarker status, while conferring smaller marginal effects in those who are already nutritionally replete, consistent with a possible “ceiling effect” in nutrient repletion models (16–18). Preliminary empirical evidence supports this repletion hypothesis. For instance, Chang et al. (19) demonstrated in a tightly controlled study that high-dose EPA significantly improved cognitive vigilance specifically in youth with low baseline endogenous EPA levels. Similarly, Gustafsson et al. reported that lower baseline EPA status was associated with favorable behavioral trajectories following supplementation (20).

Nevertheless, previous systematic reviews have not routinely incorporated this variable into pooled analyses, continuing to pool data across nutritionally heterogeneous cohorts. To address this gap, the present systematic review and meta-analysis examined whether baseline omega-3 biomarker status is associated with differential response to omega-3 supplementation in children and adolescents with ADHD. We aimed to synthesize the available biomarker-stratified RCT evidence, evaluate whether treatment effects differed between low-baseline and normal/high or unstratified baseline estimates, and exploratorily model whether continuous baseline EPA levels were associated with effect size. Given the limited number of eligible trials, these analyses were considered exploratory and hypothesis-generating.

## 2 Materials and methods

This systematic review and meta-analysis was conducted according to the updated preferred reporting items for systematic reviews and meta-analyses (PRISMA) 2020 guidelines to ensure maximal methodological transparency and rigor (21).

### 2.1 Protocol registration

The study protocol, inclusive of search strategies, eligibility criteria, and planned analytical frameworks, was prospectively registered in the International Prospective Register of Systematic Reviews (PROSPERO) database under the identification number CRD420251218861.

### 2.2 Eligibility criteria

Studies were carefully selected based on a predefined Population, Intervention, Comparison, and Outcome (PICO) framework.

#### 2.2.1 Population

Children and adolescents (aged ≤ 18 years) with a primary, formally established diagnosis of ADHD, as defined by recognized diagnostic criteria including the Diagnostic and Statistical Manual of Mental Disorders (DSM-IV or DSM-5) or the International Classification of Diseases (ICD-10 or ICD-11). No restrictions were placed on ADHD presentation subtypes, gender, or comorbidities.

#### 2.2.2 Intervention

Eligible interventions included oral supplementation with EPA, DHA, or EPA+DHA-containing formulations. Trials using omega-3/omega-6 combination products were included only when EPA and/or DHA were active components. Because the inclusion of omega-6 fatty acids such as GLA may confound the isolated effect of EPA/DHA, these trials were identified *a priori* and examined in sensitivity analyses.

#### 2.2.3 Comparison

An appropriate, visually and organoleptically matched placebo (e.g., olive oil, sunflower oil, or medium-chain triglycerides) administered under identical double-blind conditions.

#### 2.2.4 Outcomes

The primary synthesis included ADHD-related outcomes reported in biomarker-stratified RCTs, encompassing behavioral symptom ratings, cognitive performance, and academic/functional outcomes. Because these outcomes measure related but non-identical clinical domains, outcome types were coded *a priori* and examined through subgroup and exploratory moderator analyses. Studies were required to report objective baseline omega-3 biomarkers and to provide efficacy outcomes that were stratified, correlated, or otherwise separable according to baseline biomarker status.

#### 2.2.5 Classification of baseline omega-3 nutritional status

Baseline omega-3 status was classified according to the definitions used in each original trial rather than by applying a universal *post-hoc* threshold across studies. This approach was necessary because the included trials measured baseline omega-3 status using different biological matrices or indices, including erythrocyte, plasma, serum, whole blood, and combined EPA/DHA measures, and used study-specific thresholds such as absolute cut-offs or cohort-specific medians, tertiles, or quartiles. Therefore, the term “low baseline” in this review refers to a study-defined low omega-3 status subgroup within each trial, rather than to a single directly comparable biochemical deficiency threshold across trials.

Subgroups were categorized as “low baseline” only when participants were explicitly identified by the original investigators using absolute thresholds or cohort-specific splits. Studies that did not provide a separable low-baseline subgroup but reported biomarker-linked or whole-cohort efficacy data were categorized as “normal/high or unstratified” for the categorical synthesis. This comparator category should not be interpreted as uniformly omega-3 replete, but rather as estimates not meeting a separable study-defined low-baseline criterion. All subgroup definitions, biomarker matrices, thresholds or baseline values, subgroup sample sizes, stratification type, and extracted effect sizes are reported in Supplementary Table S4. Because low-baseline definitions varied across trials, direct cross-study comparability of baseline categories is limited.

### 2.3 Study design

Only parallel-group or crossover Randomized Controlled Trials (RCTs) were eligible. Observational studies, cross-sectional analyses, case reports, and non-randomized open-label trials were strictly excluded to minimize confounding bias. Additional exclusion criteria were formulated to isolate the specific effects of omega-3s: (1) studies employing broad-spectrum multinutrient interventions where the isolated effect of PUFAs could not be determined; (2) studies in distinct clinical populations where ADHD was secondary to other major medical conditions. Furthermore, for crossover trials, data were extracted exclusively from the initial parallel-group phase to circumvent the established risk of complex metabolic carryover effects inherent to lipid interventions (22, 23).

#### 2.3.1 Information sources and search strategy

A comprehensive and systematic literature search was executed across four major electronic databases: PubMed (MEDLINE), Embase, PsycINFO, and the Cochrane Central Register of Controlled Trials (CENTRAL). The final search was conducted on 8 December 2025. The complete search strategies for all four databases are provided in Supplementary Table S1. No linguistic or geographic restrictions were imposed. The search syntax was constructed using a combination of Medical Subject Headings (MeSH) and free-text keywords, adapted iteratively for each database's specific search interface. Trial registry and grey-literature records retrieved through CENTRAL and database-indexed registry entries were screened. Reference lists of included studies and relevant prior reviews were also manually checked for potentially eligible reports.

### 2.4 Study selection and data extraction

All citations retrieved from the systematic search were exported to a reference management software where duplicate records were automatically and manually expunged. The selection process operated in two distinct phases. Initially, two independent reviewers (X.F. and M.J.) screened the titles and abstracts of all unique records. Subsequently, the full texts of all articles deemed potentially eligible were retrieved and independently evaluated against the strict inclusion and exclusion criteria. Any discrepancies or interpretive conflicts between the two primary reviewers were resolved through mediated discussion with a senior third author (J.L.) acting as the final arbiter.

Data extraction was performed utilizing a pilot-tested, standardized extraction template. Two reviewers independently extracted key variables, including study characteristics, population demographics, intervention parameters, biomarker methodology, and outcome data for each defined biomarker-linked estimate, including low-baseline, normal/high-baseline, and unstratified whole-cohort estimates where applicable.

### 2.5 Quality assessment and risk of bias

The methodological quality and internal validity of the included RCTs were appraised using the revised Cochrane Risk of Bias tool for randomized trials (RoB 2) (24). Two independent researchers evaluated each trial across five distinct domains: (1) bias arising from the randomization process; (2) bias due to deviations from intended interventions; (3) bias due to missing outcome data; (4) bias in the measurement of the outcome; and (5) bias in the selection of the reported result.

### 2.6 Statistical analysis and data synthesis

Because included studies reported ADHD-related behavioral, cognitive, and academic outcomes using different measurement scales, effect sizes were standardized and pooled as Hedges' g with 95% confidence intervals (CIs). A random-effects meta-analysis framework, employing the Restricted Maximum-Likelihood (REML) estimator, was utilized *a priori* to account for expected clinical and methodological heterogeneity. Statistical heterogeneity was evaluated using the Cochran's Q test and the *I*^2^ statistic (25).

The primary investigative aim was addressed via a pre-specified categorical subgroup analysis. Data were categorized according to baseline biomarker status as low-baseline vs. normal/high or unstratified baseline estimates, as defined in Section 2.2.5 and Supplementary Table S4. Furthermore, an exploratory random-effects meta-regression analysis was conducted, utilizing baseline EPA levels to model the magnitude of the treatment effect size. All statistical meta-analyses and data visualizations were executed using the R statistical computing environment (version 4.3.1), primarily using the metafor and meta packages (26, 27). To account for potential within-study dependence where a single trial contributed multiple estimates, sensitivity analyses were conducted by retaining one effect size per parent trial according to a prespecified priority order: ADHD symptom-rating outcomes, cognitive performance outcomes, academic outcomes, and then the estimate with the smaller standard error when multiple estimates remained. Statistical significance was set at α = 0.05 (two-tailed). Publication bias and small-study effects were assessed using funnel plots. However, formal statistical assessments (e.g., Egger's test) were interpreted strictly as exploratory due to the small number of included analytical estimates (*k* = 10).

### 2.7 Certainty of evidence assessment

The certainty of evidence was evaluated using the GRADE framework (28, 29). Given that the primary synthesis relied heavily on subgroup differences, specific downgrading decisions were made regarding the *post-hoc* nature of biomarker classifications, heterogeneous biomarker matrices, study-specific baseline thresholds, heterogeneity in outcome domains, and within-study dependence.

## 3 Results

### 3.1 Search results and study selection

**Figure 1:**
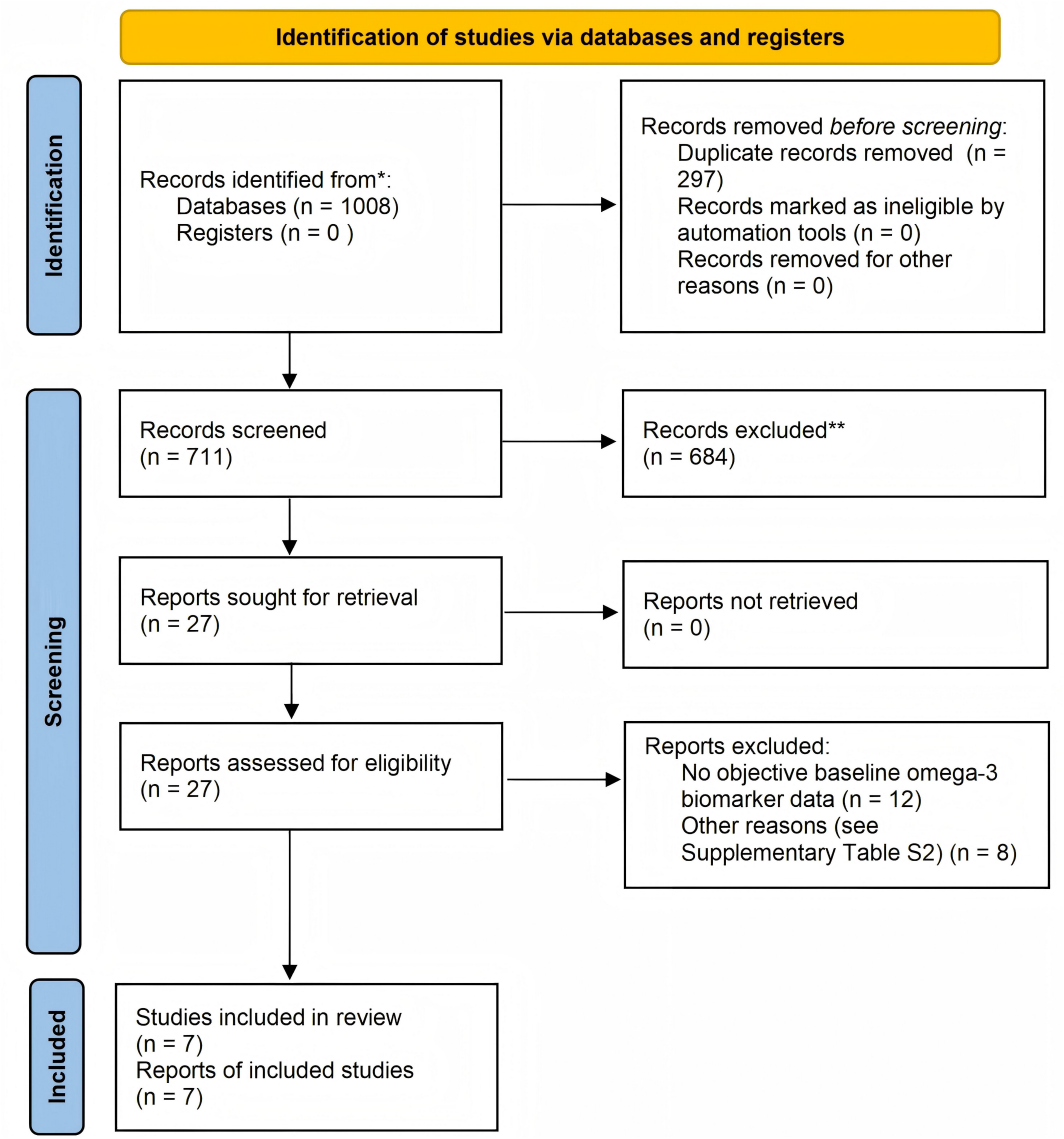
PRISMA 2020 flow diagram of study identification, screening, eligibility assessment, and inclusion. Full-text exclusion reasons are detailed in Supplementary Table S2.

The systematic literature search yielded a total of 1,008 records. Following deduplication, 711 unique titles and abstracts were screened, of which 684 were excluded. The full texts of 27 potentially eligible reports were retrieved for detailed assessment. During full-text review, 20 reports were excluded. The most common reason was absence of eligible objective baseline omega-3 biomarker-linked efficacy data, including either no baseline omega-3 biomarker measurement or no biomarker-linked efficacy outcome (*n* = 12). The remaining reports were excluded for other eligibility reasons, including wrong population or clinical condition, wrong study design, mixed or non-isolable intervention, lack of an eligible comparator, or absence of biomarker-linked efficacy outcomes (*n* = 8). Detailed full-text exclusion reasons are provided in Supplementary Table S2. The complete study selection process is shown in Figure 1.

### 3.2 Study characteristics and demographics

The 7 included RCTs provided data for 10 distinct analytical estimates (*k* = 10) owing to internal study stratifications or biomarker-linked whole-cohort analyses (19, 20, 22, 30–33). The comprehensive baseline characteristics, intervention parameters, and biomarker methodologies of these included trials are systematically detailed in Table 1. The intervention durations were clinically relevant, ranging typically from 12 to 24 weeks. Crucially, the biological matrices utilized to ascertain baseline nutritional status varied, encompassing erythrocyte membrane analysis, serum fatty acid profiles, plasma DHA fractions, and whole blood analyses. Accordingly, baseline-status categories should be interpreted as study-specific biomarker strata rather than directly comparable deficiency categories across trials.

**Table 1:** Baseline characteristics, intervention details, biomarker assessments, and ADHD-related outcome domains of included randomized controlled trials.

Study (year; country)	Sample and demographics	Medication status	Intervention and placebo	Duration	Baseline biomarker assessment	Main outcome domain
Gustafsson (2010; Sweden)	*N* = 92; mean age 9.8 years; 76% male	Drug-naïve	EPA 500 mg + DHA 2.7 mg; placebo: rapeseed oil/MCT	15 weeks	Serum EPA; low/high subgroup defined by cohort median split	Behavioral symptom rating (CTRS)
Johnson (2012; Sweden)	*N* = 75; mean age 12 years; 85% male	Drug-naïve	EPA 558 mg + DHA 174 mg + GLA 60 mg; placebo: olive oil	12 weeks	Plasma fatty-acid profile; no separable low-baseline subgroup	Behavioral symptom rating (ADHD-RS)
Milte (2012; Australia)	*N* = 90; mean age 9 years; 79% male	Drug-naïve	EPA-rich arm: EPA 1,109 mg + DHA 108 mg; DHA-rich arm: DHA 1,032 mg + EPA 264 mg; placebo: safflower oil	16 weeks	Erythrocyte EPA+DHA; low subgroup defined by lowest quartile	Academic outcome (word reading)
Widenhorn-Müller (2014; Germany)	*N* = 95; mean age 8.9 years; 78% male	Medication permitted (94% drug-naïve)	EPA 600 mg + DHA 120 mg; placebo: olive oil	16 weeks	Fatty-acid biomarker; exploratory investigator-defined cut-off	Cognitive performance (working memory)
Crippa (2019; Italy)	*N* = 50; mean age 11 years; 92% male	Drug-naïve	DHA 500 mg; placebo: wheat germ oil	24 weeks	Whole-blood DHA profile; unstratified whole cohort	Behavioral symptom rating (ADHD-RS)
Chang (2019; China)	*N* = 92; mean age 9.5 years; 86% male	Drug-naïve	EPA 1,200 mg; placebo: soybean oil	12 weeks	Erythrocyte EPA; low/high subgroups defined by tertiles	Cognitive performance (CPT)
Carucci (2022; Italy)	*N* = 160; mean age 9.7 years; 74% male	Drug-naïve	EPA 558 mg + DHA 174 mg + GLA 60 mg; placebo: olive oil	24 weeks	Whole-cohort biomarker classification; no separable low-baseline subgroup	Behavioral symptom rating (ADHD-RS)

Detailed subgroup-specific sample sizes, biomarker thresholds, reporter types, outcome time points, extracted effect sizes, and prespecified/*post-hoc* subgroup classifications are provided in Supplementary Table S4. For Milte (22), data were extracted from the initial parallel-group phase to avoid participant duplication and potential carry-over effects. ADHD-RS, ADHD Rating Scale; CPT, continuous performance test; CTRS, Conners' Teacher Rating Scale; DHA, docosahexaenoic acid; EPA, eicosapentaenoic acid; GLA, gamma-linolenic acid; MCT, medium-chain triglycerides.

### 3.3 Methodological quality and risk of bias assessment

**Figure 2:**
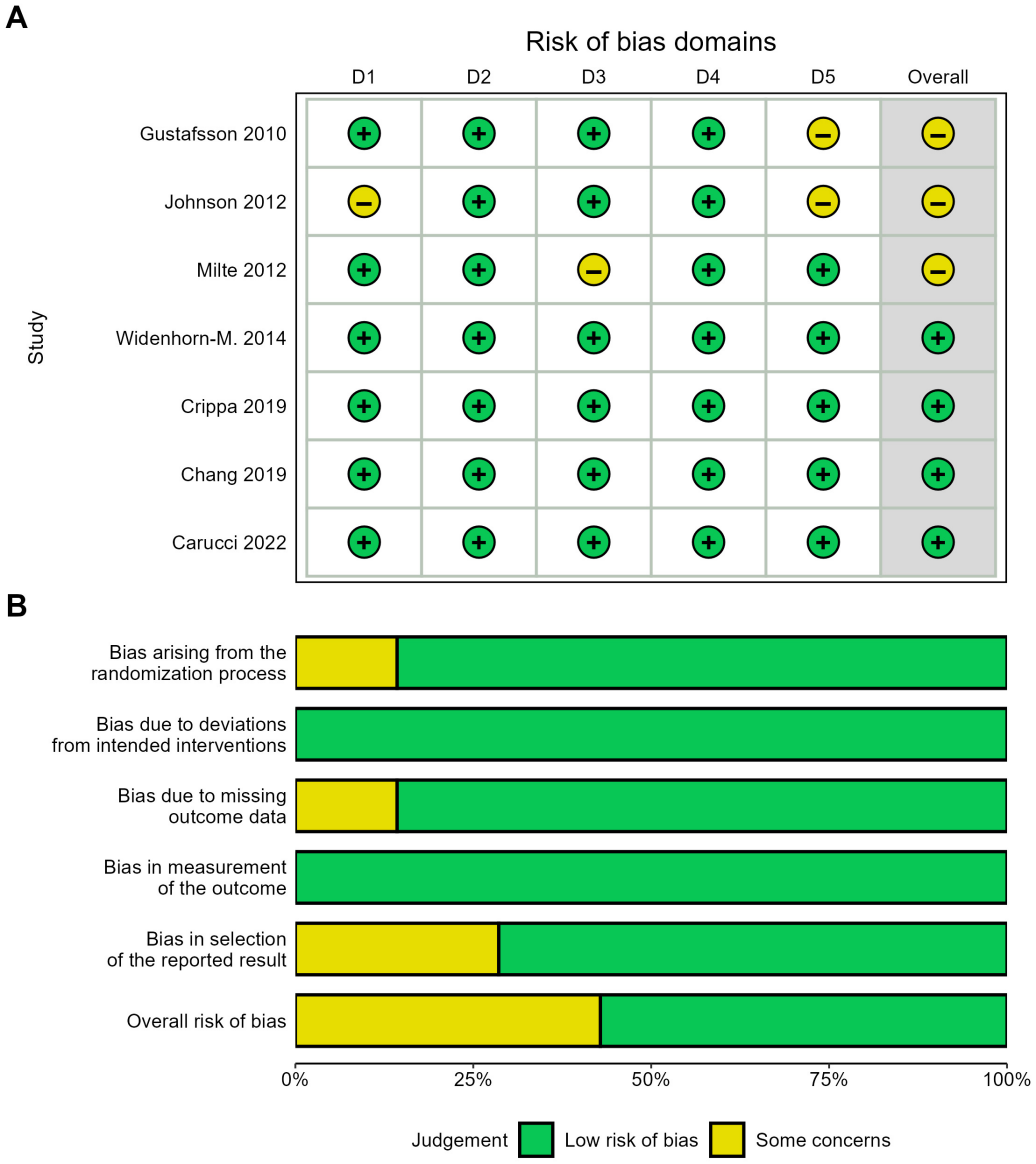
Risk of bias assessment using the revised Cochrane Risk of Bias tool for randomized trials (RoB 2). **(A)** Traffic-light plot showing domain-level judgments for each included randomized controlled trial. **(B)** Weighted summary plot showing risk-of-bias judgments across RoB 2 domains. Domain-level justifications are provided in Supplementary Table S3. D1–D5 indicate bias arising from the randomization process, deviations from intended interventions, missing outcome data, outcome measurement, and selection of the reported result, respectively.

Risk of bias was assessed using the Cochrane RoB 2 tool (Figure 2). Four studies were rated as having low risk of bias across all domains, whereas three studies were rated as having some concerns. Because only data from the initial parallel-group phase of crossover trials were extracted, no study was classified as having high risk of bias due to carryover effects.

### 3.4 Overall effect of omega-3 supplementation

**Figure 3:**
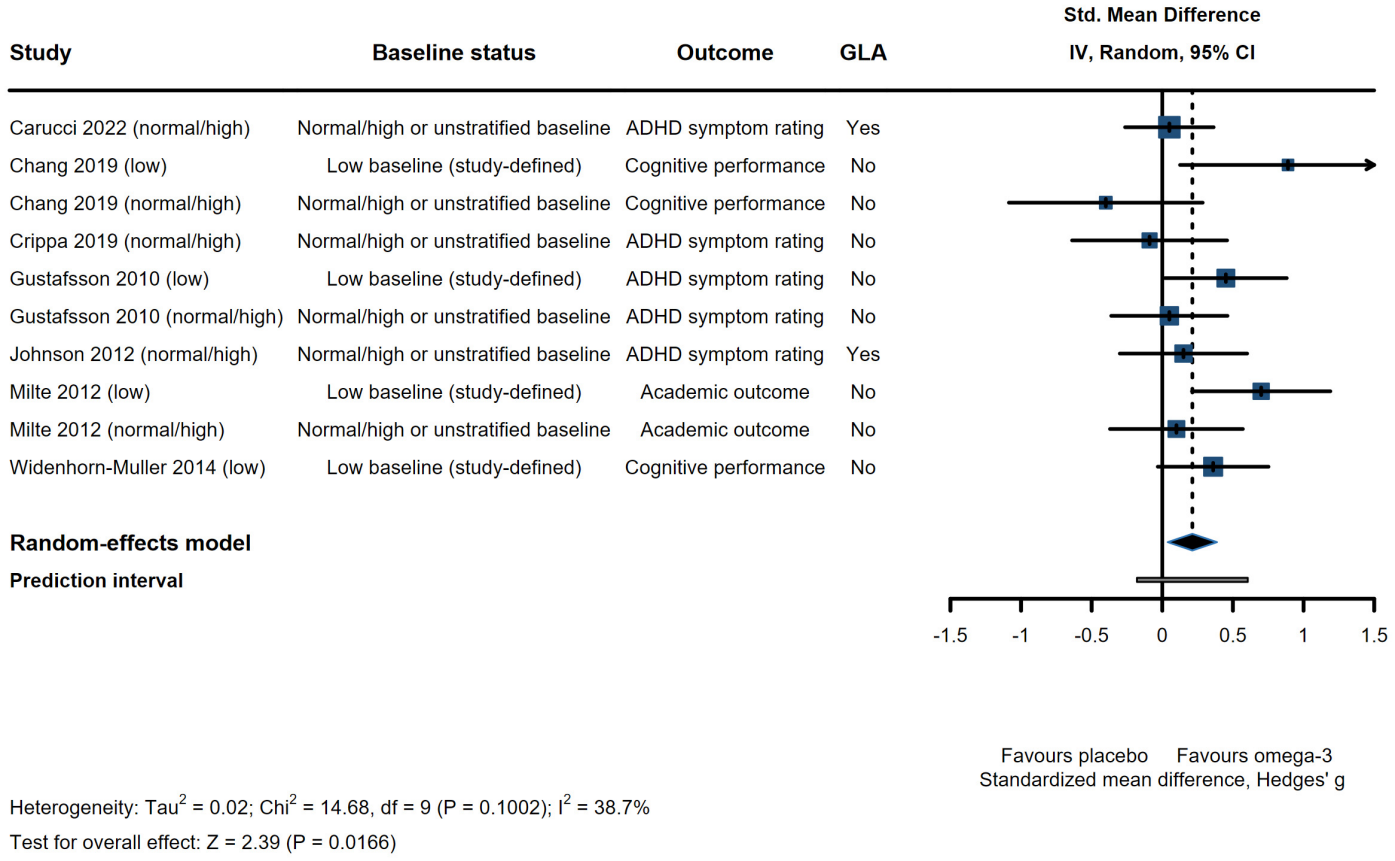
Overall random-effects forest plot of omega-3 supplementation effects on ADHD-related outcomes. Effect sizes are shown as standardized mean differences (Hedges' g) with 95% confidence intervals; positive values favor omega-3 supplementation.

When analyzing the aggregate data without accounting for baseline biomarker status, the pooled meta-analysis of all 7 RCTs (encompassing *k* = 10 distinct analytical estimates) revealed a small statistically significant overall effect of omega-3 PUFA supplementation on ADHD-related behavioral, cognitive, and academic outcomes compared to placebo. The overall standardized mean difference was 0.21 (95% CI: 0.04–0.38; *p* = 0.017). Consistent with prior systematic reviews in this field, this overall analysis demonstrated moderate observed heterogeneity (*I*^2^ = 38.7%, Cochran's Q = 14.68, df = 9, *p* = 0.1002) (Figure 3).

### 3.5 Subgroup analysis by baseline nutritional status

**Figure 4:**
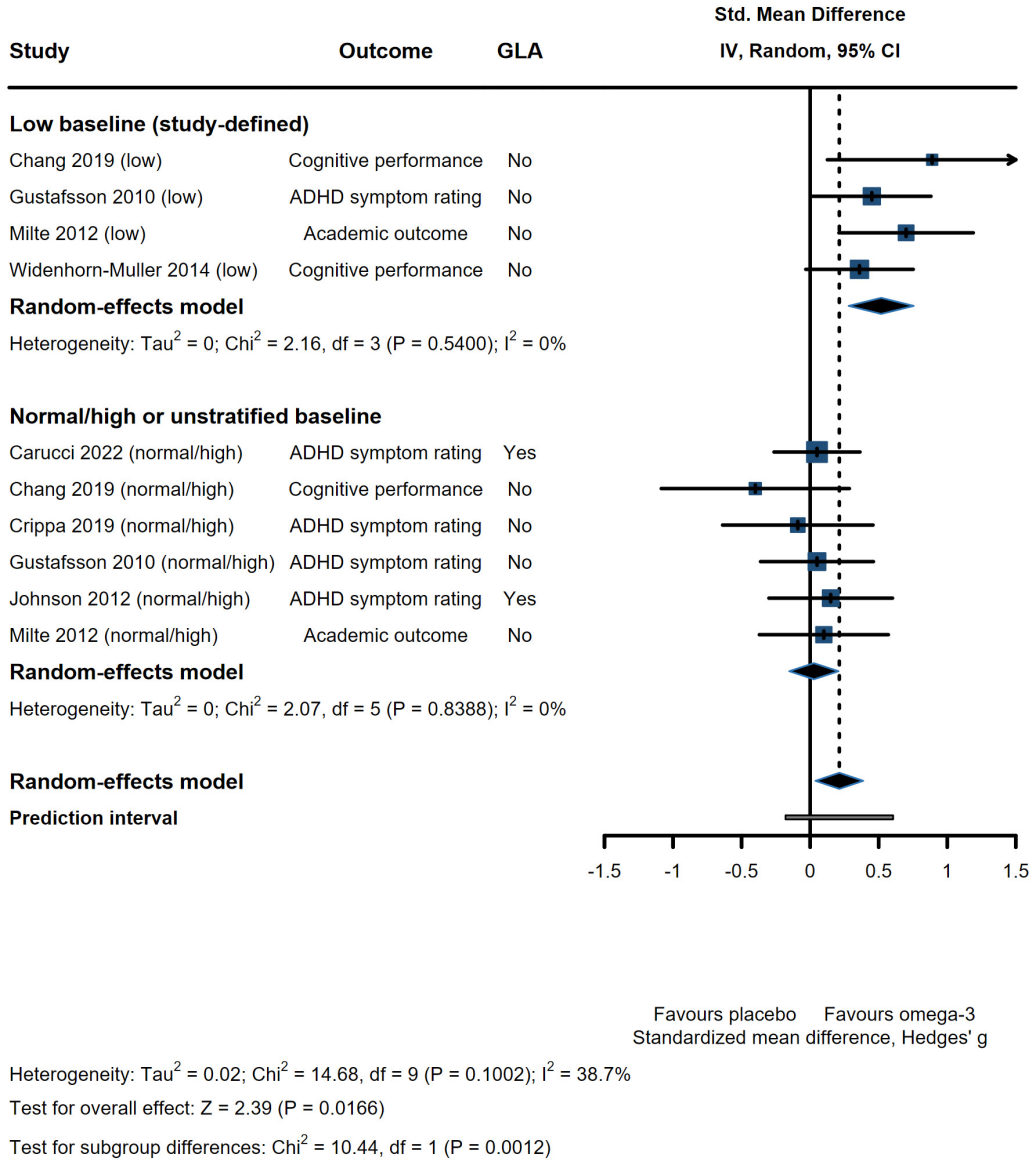
Random-effects subgroup forest plot stratified by baseline omega-3 biomarker status. Effect sizes are shown as standardized mean differences (Hedges' g) with 95% confidence intervals; positive values favor omega-3 supplementation. Estimates are grouped as study-defined low baseline versus normal/high or unstratified baseline. The test for subgroup differences indicated a statistically significant difference between baseline-status categories (χ^2^ = 10.44, df = 1, *p* = 0.0012).

Using the study-specific baseline-status definitions described above, estimates were stratified into low-baseline and normal/high or unstratified categories (Figure 4). In the primary subgroup analysis, treatment effects appeared to differ by baseline-status category (test for subgroup differences: χ^2^ = 10.44, df = 1, *p* = 0.0012), with larger estimated effects in study-defined low-baseline subgroups. However, because biomarker matrices and thresholds differed across trials, this subgroup finding should be interpreted as an exploratory signal rather than evidence based on a uniform biochemical deficiency threshold.

#### 3.5.1 Low-baseline subgroup

The low-baseline subgroup, comprising four analytical estimates (*k* = 4) classified as low baseline according to the original trial definitions, showed a larger estimated effect (SMD = 0.52, 95% CI: 0.28–0.76; *p* < 0.001). Observed statistical heterogeneity was low (*I*^2^ = 0%, *p* = 0.5400).

#### 3.5.2 Normal/high or unstratified baseline subgroup

Conversely, the normal/high or unstratified baseline subgroup (*k* = 6) did not show a statistically significant effect of supplementation (SMD = 0.03, 95% CI: −0.15–0.21; *p* = 0.77). Observed heterogeneity was low (*I*^2^ = 0%, *p* = 0.8388).

A sensitivity analysis restricted to ADHD behavioral symptom-rating outcomes (Supplementary Figure S1) yielded *k* = 5 estimates. The low-baseline estimate was numerically higher (SMD = 0.45, 95% CI: 0.02–0.88) than the normal/high or unstratified baseline estimate (SMD = 0.05, 95% CI: −0.15–0.25). However, the between-subgroup difference did not reach statistical significance (*Q* = 2.69, df = 1, *p* = 0.1009). Because only one low-baseline estimate met the symptom-rating-only criterion, this analysis was underpowered and should be interpreted as inconclusive rather than confirmatory evidence of differential efficacy.

To address the potential within-study dependence caused by parent trials contributing multiple subgroups, a multilevel random-effects model was employed. The model yielded a directionally consistent estimate (SMD = 0.21, 95% CI: 0.04–0.38; *p* = 0.016), although interpretation remains limited by the small number of parent trials and subgroup estimates. Conversely, a highly conservative sensitivity analysis retaining only one subgroup per parent trial yielded a non-significant overall effect (SMD = 0.09, 95% CI: −0.08–0.25; *p* = 0.307). This attenuation indicates that the overall pooled estimate was sensitive to analytic decisions regarding dependent subgroup estimates and should therefore be interpreted cautiously.

Furthermore, a sensitivity analysis excluding trials that utilized omega-3/omega-6 combined formulations (GLA) showed a similar subgroup pattern (Between-group *Q* = 9.51, *p* = 0.0020). In this sensitivity analysis, the estimated benefit was mainly observed in the low-baseline subgroup, whereas the normal/high or unstratified baseline subgroup showed little evidence of benefit.

### 3.6 Exploratory meta-regression

**Figure 5:**
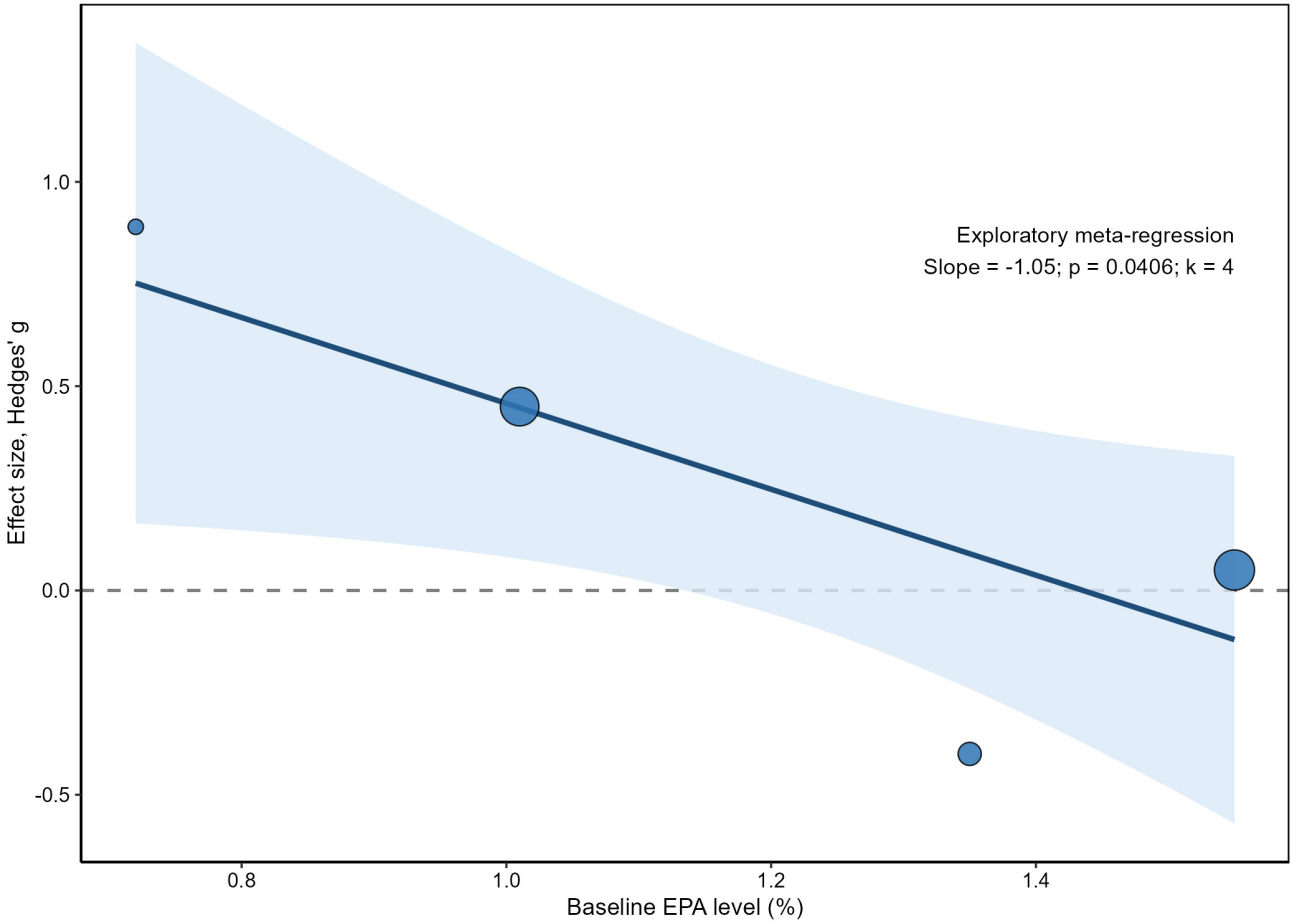
Exploratory meta-regression examining the association between baseline EPA level (%) and omega-3 supplementation effect size. Circle size reflects inverse-variance weight; the regression line and shaded area represent the fitted trend and 95% confidence interval. The analysis included four subgroups with continuous baseline EPA data and showed a nominally significant negative association (slope = −1.05, *p* = 0.0406), which should be interpreted as hypothesis-generating.

Exploratory meta-regression was possible in only four subgroups (*k* = 4) that provided continuous baseline EPA data. A nominally significant negative association was observed between baseline EPA levels and treatment effect size (slope = −1.05, *p* = 0.0406; Figure 5). However, this result must be interpreted with extreme caution because of the very small number of data points and should be considered strictly hypothesis-generating.

### 3.7 Publication bias and certainty of evidence

Visual inspection of the funnel plot (Supplementary Figure S4) did not suggest marked asymmetry; however, interpretation was limited by the small number of analytical estimates and the non-independence of some subgroup estimates from the same parent trials. Domain-level Risk of Bias justifications are provided in Supplementary Table S3. According to the GRADE framework (Supplementary Table S5), the certainty of evidence for both the low-baseline and normal/high or unstratified baseline subgroups was rated as low, primarily due to the small number of studies, *post-hoc* subgrouping, heterogeneous biomarker matrices, study-specific baseline thresholds, and heterogeneous outcome domains. Evidence from the meta-regression was rated as very low.

## 4 Discussion

This systematic review and meta-analysis represents, to our knowledge, the first comprehensive synthesis explicitly designed to evaluate whether baseline nutritional biomarkers are associated with differential response to omega-3 PUFA supplementation in pediatric ADHD. Our findings may help contextualize some of the historical inconsistencies observed in previous meta-analyses. In our initial overall pooled analysis, which ignored biomarker status, omega-3 supplementation yielded a small statistically significant average effect on ADHD-related outcomes, accompanied by moderate heterogeneity. However, this average effect was attenuated to non-significance in the one-effect-per-parent-trial sensitivity analysis (SMD = 0.09, 95% CI: −0.08–0.25; *p* = 0.307), indicating that the pooled overall estimate should be interpreted cautiously. This is consistent with prior non-stratified meta-analyses reporting modest average effects of omega-3 supplementation in pediatric ADHD (10–14). In addition, the application of biomarker stratification suggested a differential pattern, with larger effect estimates in low-baseline subgroups than in normal/high or unstratified baseline subgroups. This stratification was also associated with low observed within-subgroup heterogeneity (*I*^2^ = 0%). These findings raise the possibility that some of the mixed results in previous omega-3 ADHD literature may partly reflect heterogeneity in participants' baseline fatty-acid status, although this interpretation remains hypothesis-generating because of the small number of studies and the limitations of subgroup analyses (15, 16, 34, 35).

### 4.1 Mechanistic interpretation: the repletion model

The subgroup differences observed are consistent with a possible repletion model in nutritional psychiatry. Omega-3 PUFAs function as essential components of neuronal membranes and may influence neurodevelopmental and homeostatic processes (8, 9, 36). In children with low baseline omega-3 status (the Low Baseline subgroup), supplementation may act as a corrective nutritional intervention. EPA and DHA may integrate into phospholipid bilayers and potentially influence membrane fluidity, neurotransmission, and inflammatory signaling pathways relevant to ADHD pathophysiology (8, 9). This provides one biologically plausible mechanism, although the present meta-analysis was not designed to test mechanistic pathways directly. Omega-3s, particularly EPA and DHA, are precursors to specialized pro-resolving mediators such as resolvins and protectins, which participate in the resolution of inflammation (37, 38). Conversely, in individuals with relatively adequate baseline omega-3 status, additional supplementation may plausibly produce smaller marginal effects, consistent with a repletion-based interpretation (16–18).

### 4.2 Implications for precision-nutrition research

These findings support a precision-nutrition research framework for pediatric ADHD, particularly the development of biomarker-stratified trial designs (36, 39). This meta-analysis suggests that baseline biomarker assessment may help identify children more likely to respond to omega-3 supplementation, although this hypothesis requires confirmation in prospectively stratified trials before clinical implementation can be recommended. Validated tools for assessing omega-3 status, such as the whole-blood Omega-3 Index, may be useful in future biomarker-stratified research designs (17, 18, 40). The favorable safety profile of omega-3 PUFAs supports further evaluation as a potential adjunctive option in biomarker-defined subgroups, but current evidence is insufficient to recommend them as a routine, first-line biomarker-guided therapy. Therefore, the present findings should be interpreted as supporting future precision-nutrition research rather than immediate changes to standard clinical practice.

### 4.3 Strengths and limitations

A strength of this meta-analysis is its hypothesis-driven design and focus on objective baseline biomarker data rather than dietary recall alone. Adherence to PRISMA guidelines, prospective PROSPERO registration, and use of the Cochrane RoB 2 tool strengthened methodological transparency. However, several limitations warrant careful consideration. First and foremost is the limited volume of eligible literature. With only 7 RCTs comprehensively reporting and stratifying by biomarker data, the statistical power was constrained. Second, another important limitation concerns the operational definition of low baseline omega-3 status. The included trials did not use a harmonized biomarker matrix or a standardized biochemical threshold. Low-baseline status was defined using different matrices or indices, including serum, plasma, erythrocyte, whole-blood, or combined EPA/DHA measures, and thresholds were study-specific. Therefore, the low-baseline category should be interpreted as a study-defined relative low-status subgroup rather than a directly comparable cross-trial deficiency state. This limits the comparability of subgroup estimates and reduces the certainty with which baseline omega-3 status can be considered a clinical effect modifier. Third, and critically, the majority of baseline biomarker stratifications in the included trials were conducted as *post-hoc* analyses rather than being pre-specified. *Post-hoc* subgroup analyses are vulnerable to bias and may inflate apparent effect sizes; therefore, subgroup claims should be interpreted cautiously unless supported by prespecified hypotheses and consistent external evidence (34, 35). Accordingly, the observed subgroup effect should be interpreted as a hypothesis-generating signal rather than confirmatory evidence. Fourth, several alternative explanations for the observed subgroup differences warrant consideration. Children in the low-baseline subgroup may have had greater baseline ADHD severity (leading to regression to the mean), differing dietary patterns, or unmeasured confounders compared to replete children. Furthermore, differences in omega-3 formulation (e.g., presence of GLA) and placebo composition across trials may contribute to between-study differences. Additional alternative explanations include differences in outcome domain, omega-3 dose and EPA/DHA ratio, adherence, concomitant ADHD medication, rater type, and selective reporting. Because the low-baseline evidence base included cognitive and academic outcomes in addition to ADHD symptom ratings, the present findings should not be interpreted as definitive evidence of improvement in core ADHD symptoms alone. Finally, three of the seven included RCTs contributed two subgroups to the pooled analysis, introducing within-study dependence. A multilevel random-effects model yielded a directionally consistent estimate, but the small evidence base and the attenuation observed in the one-effect-per-parent-trial sensitivity analysis limit any firm conclusion regarding non-independence.

### 4.4 Future research directions

Future RCTs evaluating omega-3 supplementation in pediatric ADHD should consider incorporating baseline omega-3 biomarker assessment to further validate these findings. Prospectively stratified trial designs, in which participants are categorized by standardized omega-3 biomarker thresholds before randomization, would provide stronger evidence regarding whether baseline nutritional status modifies supplementation response. Standardized measures such as the Omega-3 Index may be useful for harmonizing biomarker classification across future studies (17, 18). Future studies should also prioritize clinically meaningful ADHD symptom-rating outcomes, report cognitive and academic outcomes separately, and use standardized biomarker matrices and thresholds where possible. Additional research is needed to determine whether correcting omega-3 deficiency during specific developmental periods produces clinically relevant and sustained benefits.

## 5 Conclusion

This systematic review and meta-analysis provides preliminary evidence that the response to long-chain omega-3 PUFA supplementation in pediatric ADHD may vary according to baseline omega-3 nutritional status. Larger estimated effects were observed in study-defined low-baseline subgroups, whereas normal/high or unstratified estimates showed little evidence of benefit; however, the ADHD symptom-rating-only sensitivity analysis showed only a numerically higher low-baseline estimate and no statistically significant between-subgroup difference. Because the evidence base was limited to seven RCTs, low-baseline definitions varied by biomarker matrix and study-specific threshold, and several subgroup analyses were *post hoc*, these findings should be interpreted as hypothesis-generating.

Despite these limitations, clarifying the association between omega-3 status, including EPA/DHA biomarkers, and ADHD-related outcomes in children remains clinically valuable. The present findings suggest that baseline omega-3 nutritional status may influence supplementation response and provide a potential direction for future precision-nutrition interventions. Larger, adequately powered, prospectively stratified RCTs with harmonized biomarker matrices, standardized baseline cut-offs, consistent outcome domains, and transparent subgroup reporting are needed to verify this trend and provide more reliable evidence for clinical nutritional guidance in children and adolescents with ADHD.

## Data Availability

The original contributions presented in the study are included in the article/Supplementary material, further inquiries can be directed to the corresponding authors.

## Supplementary material

Supplementary Figure S1 Random-effects forest plot restricted to ADHD symptom-rating outcomes.

Supplementary Figure S2 One-effect-per-parent-trial sensitivity analysis.

Supplementary Figure S3 Sensitivity analysis excluding GLA-containing formulations.

Supplementary Figure S4 Funnel plot for visual assessment of potential publication bias and small-study effects. Interpretation was limited by the small number of analytical estimates and by the non-independence of some subgroup estimates derived from the same parent randomized controlled trials.

Supplementary Table S1 Complete database-specific search strategies.

Supplementary Table S2 Full-text reports excluded after eligibility assessment and primary reasons for exclusion.

Supplementary Table S3 Risk of bias judgments and domain-level justifications using the revised Cochrane Risk of Bias tool for randomized trials (RoB 2).

Supplementary Table S4 Subgroup definitions, baseline biomarker thresholds, and extracted effect sizes used in the biomarker-stratified meta-analysis.

Supplementary Table S5 GRADE certainty-of-evidence assessment for biomarker-stratified omega-3 supplementation effects.
