# Supplementary figures and images for: Baseline omega-3 nutritional status and supplementation response in pediatric attention-deficit/hyperactivity disorder: a systematic review and biomarker-stratified meta-analysis
Source: Front Public Health. 2026 Jun 22;14:1844881. doi: 10.3389/fpubh.2026.1844881 (PMC13353153; doi:10.3389/fpubh.2026.1844881)

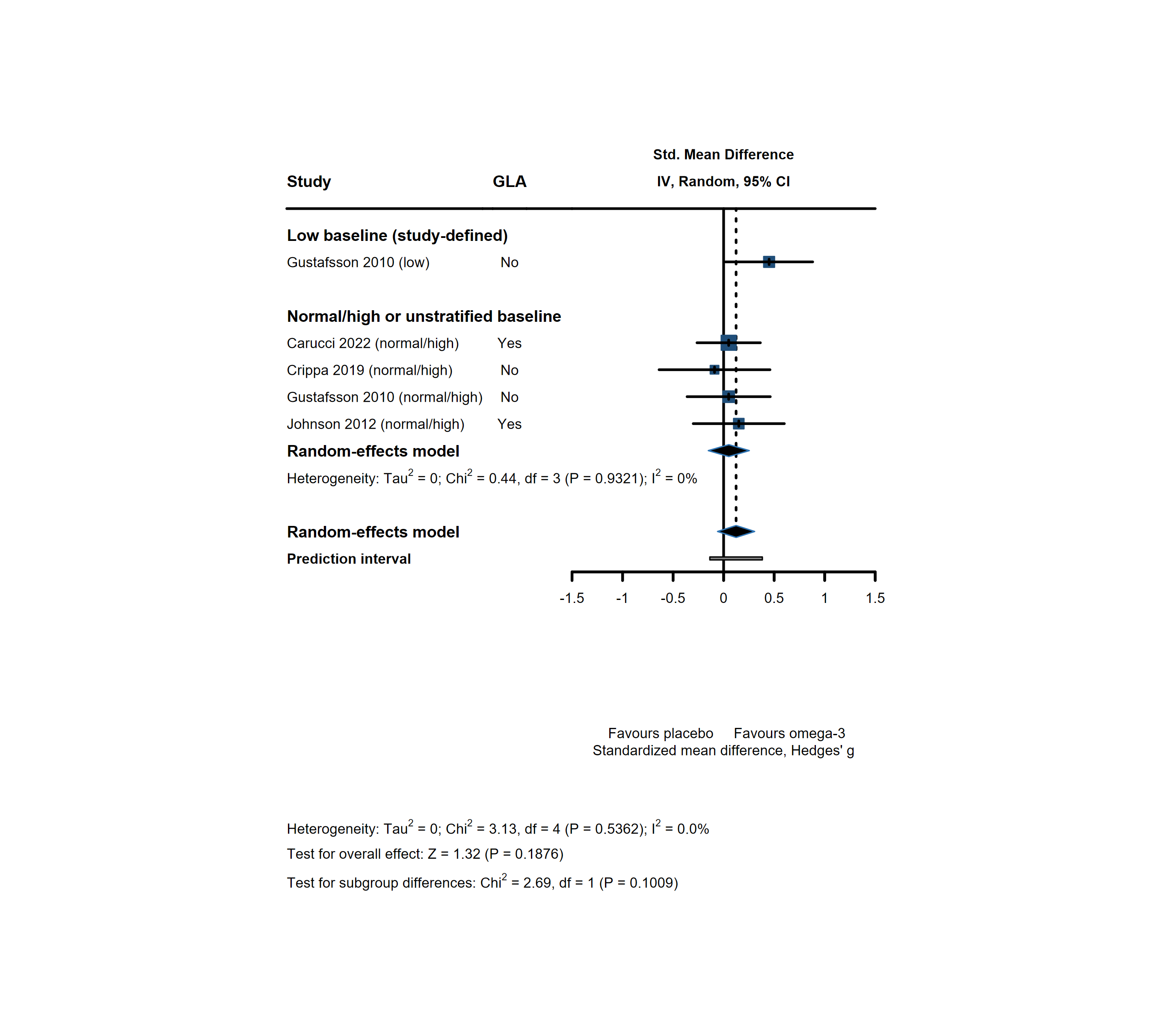

Supplement: Supplementary Figure S1 — Random-effects forest plot restricted to ADHD symptom-rating outcomes. [file Image_1.tiff]

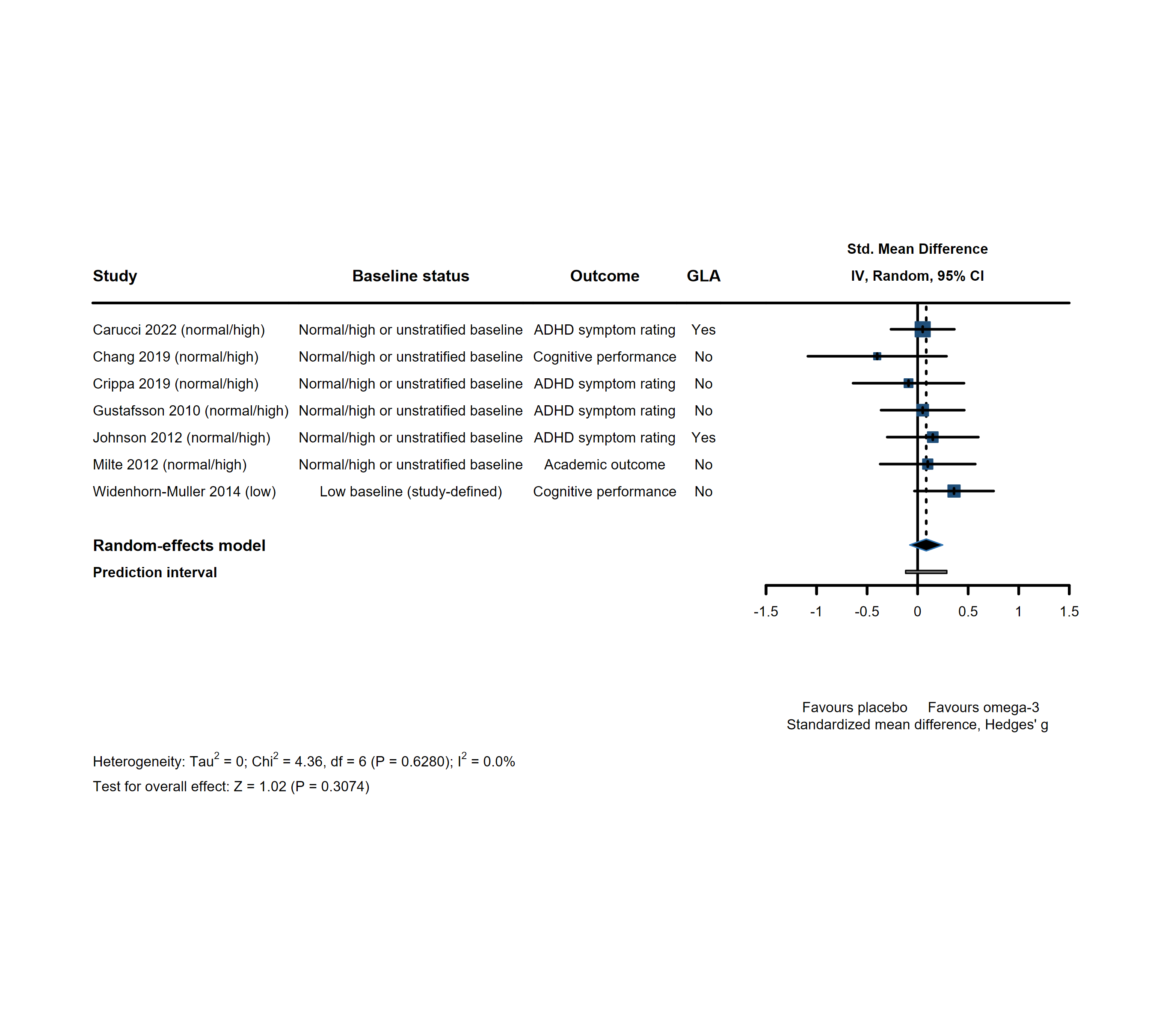

Supplement: Supplementary Figure S2 — One-effect-per-parent-trial sensitivity analysis. [file Image_2.tiff]

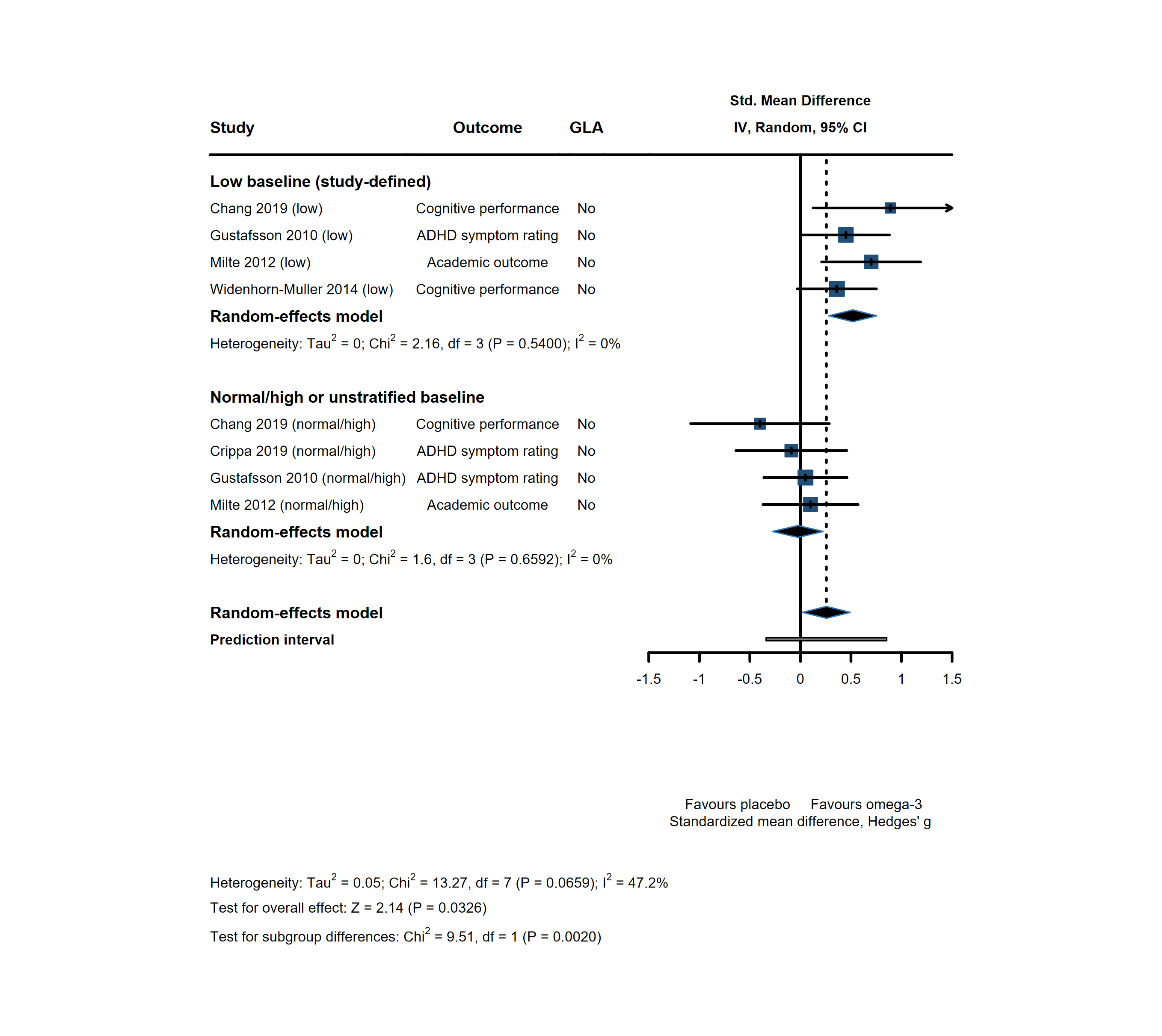

Supplement: Supplementary Figure S3 — Sensitivity analysis excluding GLA-containing formulations. [file Image_3.tiff]

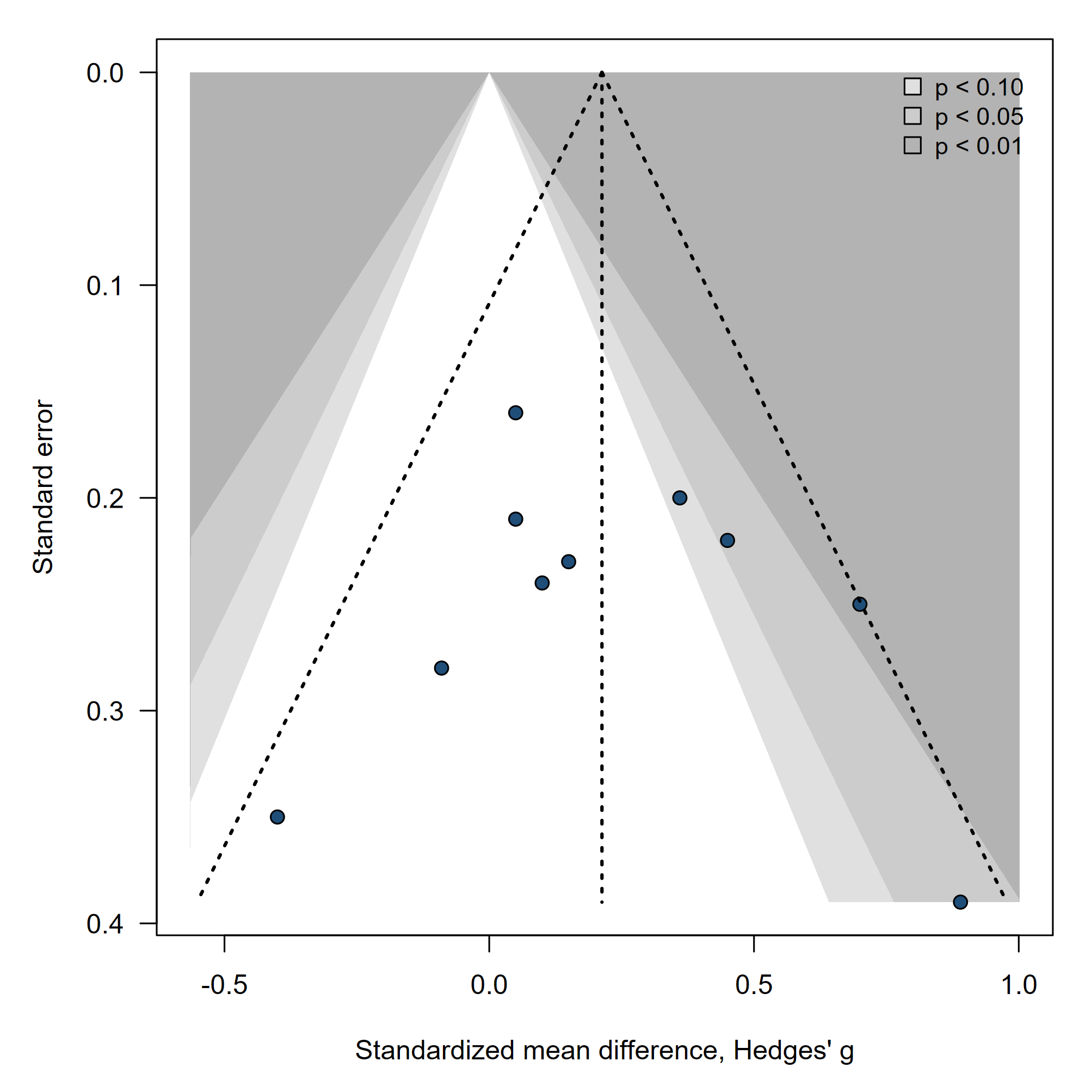

Supplement: Supplementary Figure S4 — Funnel plot for visual assessment of potential publication bias and small-study effects. Interpretation was limited by the small number of analytical estimates and by the non-independence of some subgroup estimates derived from the same parent randomized controlled trials. [file Image_4.tiff]
